# Supplementary material for: Establishing Animal Welfare Rules of Conduct for the Portuguese Veterinary Profession—Results from a Policy Delphi with Vignettes
Source: Animals (Basel). 2020 Sep 8;10(9):1596. doi: 10.3390/ani10091596 (PMC7552194; doi:10.3390/ani10091596)
Supplement: Supplementary file 1 [file animals-10-01596-s001.zip › Suplemmentary Material - Quantitative Results.docx]

**Table S1.** Quantitative results regarding the vignette on Euthanasia used in Round 2 of the Policy Delphi.

|  | **N/A** | **Strongly Disagree** | **Disagree** | **Neutral** | **Agree** | **Strongly Agree** | **TOTAL** | **Weighted Average** | **Standard Deviation S** |
| --- | --- | --- | --- | --- | --- | --- | --- | --- | --- |
| The vet's conduct is justified under the circumstances. | 4.88% | 0.00% | 12.20% | 4.88% | 36.59% | 41.46% | 100.01% | 4.13 | 1.00 |
| The vet took the best possible decision. | 4.88% | 0.00% | 17.07% | 0.00% | 48.78% | 29.27% | 100.00% | 3.95 | 1.02 |
| The vet acted in the best interest of the public. | 2.44% | 4.88% | 9.76% | 12.20% | 34.15% | 36.59% | 100.02% | 3.9 | 1.17 |
| The vet acted in the best interest of the animal. | 2.44% | 7.32% | 9.76% | 12.20% | 39.02% | 29.27% | 100.01% | 3.75 | 1.21 |
| The vet should have handed the cat to an animal charity. | 2.44% | 24.39% | 34.15% | 26.83% | 12.20% | 0.00% | 100.01% | 2.28 | 0.99 |
| The vet's conduct constitutes a disciplinary offense. | 4.88% | 41.46% | 31.71% | 17.07% | 4.88% | 0.00% | 100.00% | 1.85 | 0.90 |
| The vet's conduct must be punished by the OMV. | 7.32% | 56.10% | 29.27% | 7.32% | 0.00% | 0.00% | 100.01% | 1.47 | 0.65 |
| Average | 4.18% |  |  |  |  |  |  |  |  |

**Table S2.** Quantitative results regarding the vignette on Fitness for Transport used in Round 2 of the Policy Delphi.

|  | **N/A** | **Strongly Disagree** | **Disagree** | **Neutral** | **Agree** | **Strongly Agree** | **TOTAL** | **Weighted Average** | **Standard Deviation S** |
| --- | --- | --- | --- | --- | --- | --- | --- | --- | --- |
| The vet acted in the best interest of the farmer. | 4.88% | 0.00% | 2.44% | 0.00% | 56.10% | 36.59% | 100.01% | 4.33 | 0.62 |
| The vet's conduct is justified under the circumstances. | 2.44% | 7.32% | 9.76% | 14.63% | 34.15% | 31.71% | 100.01% | 3.75 | 1.24 |
| The vet took the best possible decision. | 4.88% | 7.32% | 17.07% | 17.07% | 26.83% | 26.83% | 100.00% | 3.51 | 1.30 |
| The vet acted in the best interest of the public. | 2.44% | 2.44% | 21.95% | 36.59% | 19.51% | 17.07% | 100.00% | 3.28 | 1.09 |
| The vet acted in the best interest of the animal. | 2.44% | 14.63% | 29.27% | 24.39% | 9.76% | 19.51% | 100.00% | 2.9 | 1.35 |
| The vet's conduct constitutes a disciplinary offense. | 9.76% | 26.83% | 24.39% | 14.63% | 19.51% | 4.88% | 100.00% | 2.46 | 1.28 |
| The vet's conduct must be punished by the OMV. | 9.76% | 29.27% | 31.71% | 14.63% | 9.76% | 4.88% | 100.01% | 2.22 | 1.18 |
| Average | 5.23% |  |  |  |  |  |  |  |  |
