# Supplementary figures and images for: Establishing Animal Welfare Rules of Conduct for the Portuguese Veterinary Profession—Results from a Policy Delphi with Vignettes
Source: Animals (Basel). 2020 Sep 8;10(9):1596. doi: 10.3390/ani10091596 (PMC7552194; doi:10.3390/ani10091596)

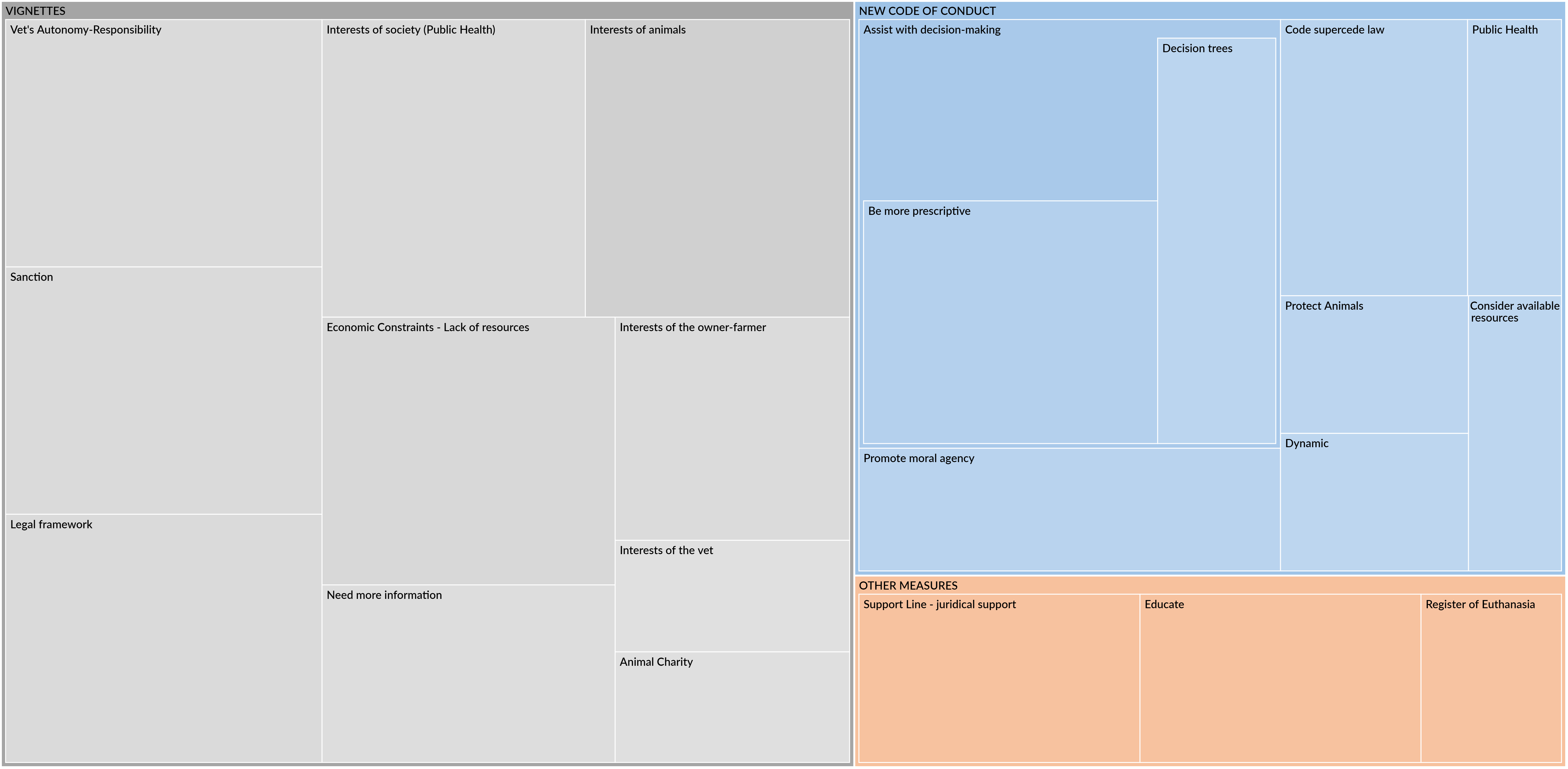

Supplement: Supplementary file 1 [file animals-10-01596-s001.zip › Figure S1.png]
